# Lipid Droplets Accumulation in the Brain of HIV Transgenic Rat: Implication in the Accelerated Aging of HIV Infected Individuals

**DOI:** 10.14336/AD.2024.0125

**Published:** 2024-01-25

**Authors:** Ming-Lei Guo, Yan Cheng, Damian Martinez Pineda, Rachael E. Dempsey, Lifang Yang

**Affiliations:** ^1^Drug Addiction Laboratory, Department of Pathology and Anatomy, Eastern Virginia Medical School, Norfolk, Virginia, USA.; ^2^Leroy T. Canoles Jr. Cancer Research Center, Department of Microbiology and Molecular Cell Biology, Eastern Virginia Medical School, Norfolk, Virginia, USA.; ^3^Center for Integrative Neuroscience and Inflammatory Diseases, Eastern Virginia Medical School, Norfolk, Virginia, USA.

**Keywords:** lipid droplet, microglia, neuroinflammation, HIV, aging

## Abstract

Abnormal microglial activation has been suggested as “driven force” promoting brain aging. Lipid droplets accumulating microglia (LDAM), identified as a novel inflammatory phenotype, elevate neuroinflammation and exaggerate neuronal injuries in aging and multiple neurodegenerative diseases. Since chronic HIV (human immunodeficiency virus) (+) individuals show an accelerated brain aging and higher incidence of neurological symptoms compared to age-matched HIV (-) population, we hypothesize that LDAM are also involved in such phenomenon. For validating the hypothesis, we employed HIV transgenic (HIV-Tg) and wilt type (WT) rats to check lipid droplets (LDs) accumulation in the brains at mature (6 months) and middle age (12 months). Our results showed that HIV-Tg rats possess higher levels of LDs formation in the hippocampus (HP) and prefrontal cortex (PFc) than controls at middle age. Increased LDs are mainly presented in microglia in the HP but largely co-localized with astrocytes in the PFc. Interestingly, increased LDs are associated with upregulation on Iba1 but not with GFAP levels. HIV-Tg rats reveal an accelerated LDs accumulation during normal aging. Purified microglia from HIV-Tg rats (12 month) show higher expression of neuroimmune signaling than microglia from controls. HIV-Tg rats showed dysregulation on cholesterol synthesis in the brain HP as well as deficiency on locomotion coordination compared to controls. Overall, our results demonstrate substantial LDs accumulation in the brains of HIV-Tg rats which is associated with abnormal microglial activation and accelerated decline on locomotion coordination during aging. Dysregulation on lipid metabolism might underlie accelerated brain aging in the context of chronic HIV infection.

## INTRODUCTION

In the era of cART (combined antiretroviral therapy), the life-expectancy of persons living with human immunodeficiency virus (PLWHIV) has been significantly increased to a comparable level with HIV (-) population. However, their life-quality has been still deeply compromised due to about 50% of PLWHIV diagnosed with HIV-associated neurological disorders (HAND) [1-3]. PLWHIV also show accelerated brain aging process [4, 5]. Another most common comorbidity observed in PLWHIV is HIV-associated lipodystrophy (HAL) with worldwide prevalence of 13 - 70% in the early 2000s [6-8]. HAL is characterized by the lipoatrophy at face, arms, and legs and lipohypertrophy at truncal areas [9-11]. One facet of lipohypertrophy is ectopic fat depots into various tissues including the liver, muscle, and cardiovessels [12-16]. Such alteration on fat distribution (i.e., lipid metabolism dysregulation) has been well-known for increasing the expression of various inflammatory markers in peripheral systems which promote insulin resistance, obesity, and atherosclerosis in PLWHIV [17-19]. However, whether lipid metabolism could be also dysregulated in the brains and such dysregulation could be linked with neurological symptoms observed in PLWHIV remains much unexplored.

Abnormal lipid/cholesterol metabolism and subsequent LDs accumulation in the brain have been paid increasingly attention in normal aging process as well as in multiple neurodegenerative diseases [20-22]. At physiological conditions, LDs provide substrates for energy metabolism, biological membranes, signaling molecules, and function as a buffering system against lipotoxicity [23, 24]. At normal condition, LDs formation is at low levels in neuroglia but can be accumulated during aging or in pathologic states (i.e., neurodegenerative diseases, cancer, stroke) [25-28]. Microglial LDs can either protect the brain [28] or contribute to the brain disorders [29]. Recently, a novel phenotype of microglia called lipid droplet-accumulating microglia (LDAM) has been identified in mouse models mimicking human neurodegenerative disease [22]. LDAM are characterized with phagocytosis defection, excessive secretion of proinflammatory cytokines, high levels of reactive oxygen species (ROS), and cholesterol efflux reduction [22, 30, 31]. LDAMs have been identified in multiple neurogenerative disorders reviewed in [32, 33]. Increased LDs formation serve as a structural marker of inflammation [34] and are linked with immune responses in macrophage [35, 36] and in Mg [37, 38].

LDs are dynamic intracellular organelles mainly composed of lipid and cholesterol and LDs formation is coordinately regulated by several different steps including the synthesis, lipid influx and efflux, and degradation. Mechanistically, sterol regulatory element-binding protein (SREBPs) family is responsible for the synthesis and highly conserved among species [39, 40]. SREBP1a and SREBP1c mainly undertake fatty acid metabolism and SREBP2 is primarily for cholesterols [41]. As a master regulator of cholesterol synthesis, SREBP2 increases the expression of several molecules including Hydroxymethylglutaryl- (HMG) coenzyme A (CoA) reductase (HMGCR), HMG-CoA synthase, mevalonate kinase and LDL receptor (LDLR). At subcellular location, SREBPs are mainly located in the endoplasmic reticulum (ER) membrane. In the condition of limited cholesterols, SREBPs could be upregulated and translocated into Golgi apparatus where they are cleaved by two proteases (site-1 and site-2 proteases) releasing the amino-terminal SREBPs. Followed, the released SREBPs enter the nucleus binding to the promoter regions of downstream genes for their active transcription [39]. In addition to the synthesis pathway, LDs formation is also regulated by lipophagy and lipoprotein lipase in microglia [31, 42].

Immune responses are heavily involved in lipid metabolism and LDs accumulation. Lipopolysaccharide (LPS) could increase LDs formation in microglia in a time-dependent course *in vitro* and c-Jun N-terminal kinase (JNK) and p38 MAPK signaling are associated with this alteration [28]. LPS also increases LDs formation in olfactory bulb microglia *in vivo* accompanying with TLR2 (toll-like receptor 2) and CD (Cluster of Differentiation) 68 upregulation [44]. HIV proteins have been shown to have the ability to dysregulate lipid metabolism. HIV transactivator of transcription (TAT) is capable of dysregulating cholesterol homeostasis in neurons and astrocytes which can be linked with HAND parthenogenesis [45, 46]. HIV-TAT could increase lipid peroxidation and dysregulate lipid profile in microglia *in vitro* [47]. Another HIV protein, negative regulatory factor (Nef), has been well-known its ability to interrupt cholesterol metabolism and the formation of lipid raft *in vivo* [48-50]. Of note, clinical studies have revealed that SREBP2, HMGCR, and LDLR are dysregulated in PLWHIV compared to HIV- population [43]. However, there is no direct/solid evidence showing that HIV proteins could regulate lipid metabolism in microglia *in vivo* till now.

In this study, we hypothesized that HIV-Tg rats would show accelerated LDs formation in the brain comparing to age-matched WT counterparts during aging. For testing this idea, we employed HIV-Tg and WT rats to explore the effects of HIV proteins on lipid metabolism in the brains during normal aging. Our results demonstrate that HIV-Tg rats show higher levels of LDs in the brain than WT rats do at middle age and such LD increase is region-specific. Meanwhile, LDs accumulation is associated with abnormal microglial activation and locomotion coordination deficiency in HIV-Tg rats. These findings suggest that lipid dysregulation might underlie accelerated brain aging and could be a novel target for ameliorating neurological symptoms in the context of chronic HIV infection.

## MATERIALS AND METHODS

### Animals

HIV-Tg and wild type (WT) rats were maintained in animal facility of Eastern Virginia Medical School. They were housed and kept in a colony room with food and water available ad libitum. The colony room was maintained on a 12:12 light to dark cycle and ambient temperature at 24.0°C ± 1.5°C. All procedures were conducted in accordance with the National Institutes of Health’s Guide for the Care and Use of Experimental Animals and were approved by Eastern Virginia Medical School’s Institutional Animal Care and Use Committee (protocol number: 23-009). BODIPY™ 493/503 was purchased from Thermo Scientific (D3922) and dissolved in DMSO at stock concentration of 2 mM. The working solution was diluted to 2 µM.

### Adult microglia isolation

HIV-Tg and WT rats were anesthetized with 4% isoflurane and transcardially perfused with 1X PBS followed by brain removal. The brain hippocampi were pooled for adult microglia isolation based on the recommend protocol (MACS dissociation kits, Miltenyi Biotech Company, Bergisch Gladbach, Germany). Briefly, brains were homogenized in 2 mL enzyme mixture by using a gentleMACS™ Octo Dissociator at 37 ^0^C for 30 min. The homogenates were then transferred to MACS® Smart Strainer followed with centrifuging. The pellets were processed for debris and red blood cell removal and dissolved in 500 µL labelling solution with CD11b beads. The purified microglia were suspended in 1 mL 1X PBS with 0.5% BSA and stored in a freezer at -80 ^0^C for later biochemical analysis. For quality check, we seeded adult microglia into 24-well plates for Ib1 immunostaining (> 90% positive).

### Rat inflammation array analysis

Applied Biosystems TaqMan™ Array (rat) 96-well Plate (Thermo Scientific, Cat # 4414081) contains four classes of inflammatory-related genes: channels, enzymes and inhibitors, factors, and receptors. Around 100 ng RNA extracted from adult microglia (HIV Tg and WT rats) were applied into the plates based on the recommended protocol (SuperScript™ III Platinum™ One-Step qRT-PCR Kit, Invitrogen). The relative expression levels (HIV-Tg vs WT) were calculated based on internal controls set in the plate. We employed integrated pathway analysis (IPA) to identify the relevant lipid metabolism pathways.

### Western blots

Brain tissues were dissolved in RIPA buffers followed with standard protein extraction procedure. After BCA quantification, equal amounts of the proteins (25 µg) were electrophoresed in SDS gels followed by transfer to PVDF membranes. The membranes were blocked by TBST then incubated with indicated antibodies overnight at 4°C. The next day, the membranes were washed and incubated with appropriate IRDye fluorescent mouse or rabbit second antibody for one hour. After three washes, the membranes were put into the Odyssey® Imaging System for image development and the intensity of fluorescent band were quantified using Image Studio™ Software. After imaging, the membranes were re-probed by β-actin for normalization. The following antibodies were used at the indicated concentration in our studies: SREBP1 (1: 2000, Novus Biological, NB100-2215), SREBP2 (1: 2000, Novus Biological, NB100-74543); HMGCR (1: 2000, Novus Biological, NBP2-66888); PLIN2 (1: 2000, Proteintech, 15294-1-AP); beclin1 (1: 2000, NB500-249), LC3B (1: 2000; NB100-2220), p62(1: 2000; Novus Biological, H00008878-M01); APP (1: 2000, Sigma, SAB520-0113); beta-amyloid (1: 2000, Sigma, AB5078P), Tau (1: 20000, Sigma, T9450); β-actin (Santa Cruz; 1: 2000, sc-8432) or (Sigma; 1: 2000, A2066). Second antibodies were purchased from Li-COR company: IRDye® 680RD Donkey anti-Mouse (1: 5000, P/N: 926-68072) and rabbit IgG; IRDye® 800CW Donkey anti-Mouse or rabbit IgG (1: 5000, P/N: 926-32213).

### Brain cryosection and double immunofluorescence staining

Rats were anesthetized with 4% isoflurane and transcardically perfused with 1X PBS followed fixed with 4% PFA. The brains were removed and put into 30% sucrose PBS solution in the refrigerator. One day later, the brain sections were cut at 30 µM. Brain sections were incubated with primary anti-AIF1 antibody (1: 1000, Wako Pure Chemical Industries, Osaka, Japan, 019-19741) or anti-GFAP antibody (1: 1000, abcam, ab7260) overnight at 4°C. Secondary AlexaFluor 488 goat anti-rabbit IgG (A-11008) or AlexaFluor 594 goat anti-mouse (A-11032) (Thermo Fisher Scientific Waltham, MA, USA) was added for 2 h incubation. Then the sections were incubated with Bodipy solution (2 µM) for 30 min at room temperature. After washing, the sections were mounted with prolong gold antifade reagent with 4,6-diamidino-2-phenylindole (Thermo Fisher Scientific, Waltham, MA, USA, P36935). Fluorescent images were acquired on a Zeiss Observer. Zenpro software (Carl Zeiss, Thornwood, NY, USA) and ImageJ were used to process and analyze the intensity Iba1/GFAP signals and lipid droplets. For the quantification of Iba1 or GFAP, two slices picked from per rats (three rats/group) were imaged and quantified under identical exposure conditions (20 X magnitude). All Iba1+ or GFAP+ cells and LDs were detected based on the threshold fluorescence intensity of each cell (ImagJ) within the counting frame of the interested regions. Then, the mean intensity of each soma was measured, and data were compared between HIV-Tg and control group. The co-localization of LDs with Iba-1 or GFAP was calculated manually on above 100 cells selected at six fields from three rats. Secondary antibody only controls were used to distinguish staining from background.

### Rotarod tests

Before testing, the rats were trained for two days to learn how to balance on the rotarod (Bioseb Company) at a speed of 4 rpm. On the test day, the rats (n = 8) were put on the rotarod which was set to accelerate from 4 to 40 rpm over 5 min. Three trials were conducted and the average latency for each rat to fall off the rotating shaft was recorded. The apparatus was cleaned with 70% ethanol between trials. Fall latencies were compared amongst groups of rats as a measure of locomotor coordination.

### Statistical analysis

All data are expressed as means ± the standard error of the mean (SEM). Data were statistically evaluated using non-parametric two-tailed t-test using GraphPad Prism 8 (La Jolla, CA, USA). The figures were produced by GraphPad prism 8. Tests with probability levels of < 0.05 were considered statistically significant. For behavioral tests, each group included at least six to eight rats for analysis.

**Figure 1.**
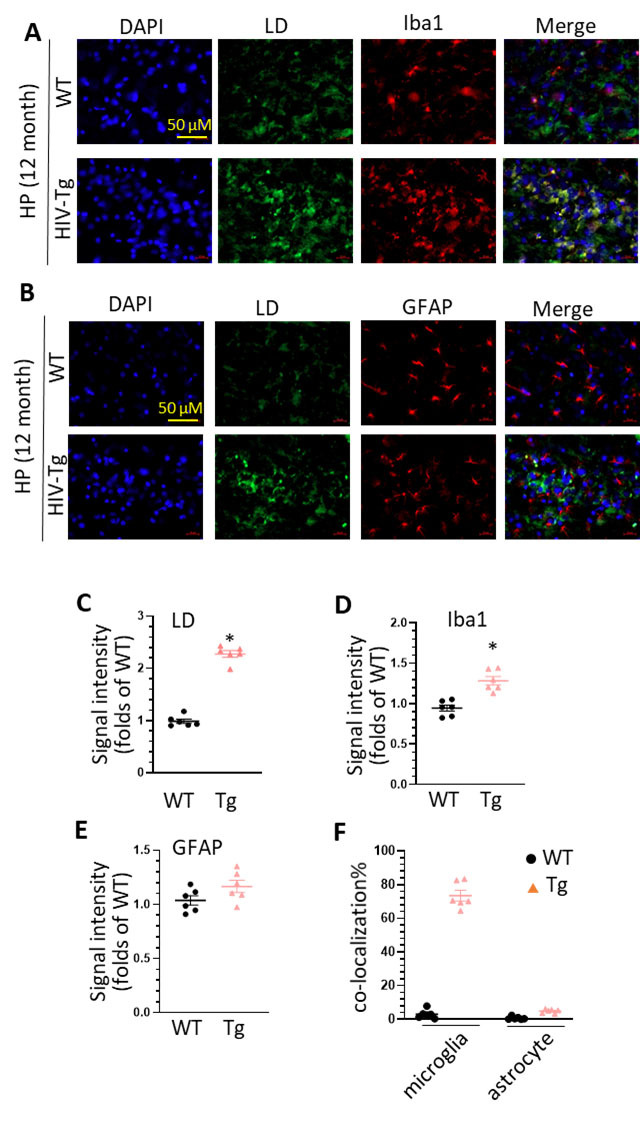
**LDs accumulation in the HP of middle age of WT and HIV-Tg rats**. (**A**) increased LDs accumulation and co-localization of LDs and Iba1 staining in the HP of WT and HIV-Tg rats (scale bar = 50 µm); (B) increased LDs accumulation and co-localization of LDs and GFAP staining in the HP of WT and HIV-Tg rats (scale bar = 50 µm); (C) statistical analysis of LDs signal intensity in the HP of WT and HIV-Tg rats (* P < 0.05); (D) statistical analysis of Iba1 signal intensity in the HP of WT and HIV-Tg rats (* P < 0.05); (E) statistical analysis of GFAP intensity in the HP of WT and HIV-Tg rats (* P < 0.05); (F) statistical analysis of the co-localization of LDs with Iba1 and GFAP in the HP of WT and HIV-Tg rats. Each group contains 3 rats and two slices were selected from each rat (n = 6), unpaired two tail t-test was used for statistical analysis.

**Figure 2.**
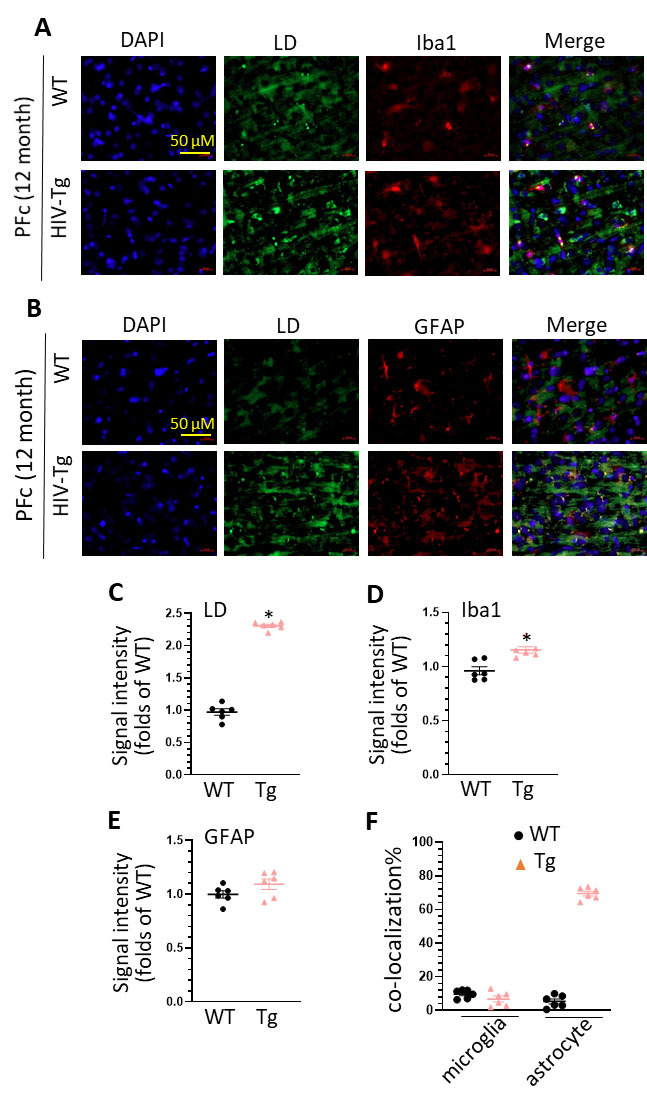
**LDs accumulation in the PFc of middle age of WT and HIV-Tg rats**. (**A**) increased LDs accumulation and co-localization of LDs and Iba1 staining in the PFc of WT and HIV-Tg rats (scale bar = 50 µm); (B) increased LDs accumulation and co-localization of LDs and GFAP staining in the PFc of WT and HIV-Tg rats (scale bar = 50 µm); (C) statistical analysis of LDs signal intensity in the PFc of WT and HIV-Tg rats (* P < 0.05); (D) statistical analysis of Iba1 signal intensity in the PFc of WT and HIV-Tg rats (* P < 0.05); (E) statistical analysis of GFAP intensity in the PFc of WT and HIV-Tg rats (* P < 0.05); (F) statistical analysis of the co-localization of LDs with Iba1 and GFAP in the PFc of WT and HIV-Tg rats. Each group contains 3 rats and two slices were selected from each rat (n = 6), unpaired two tail t-test was used for statistical analysis.

## RESULTS

### HIV-Tg rats show enhanced LDs formation in microglia in the hippocampus (HP)

To explore the long-term effects of HIV proteins on LDs formation *in vivo*, the brains of HIV-Tg and control rats (both genders, 12-month, n = 3) were removed out for cryo-section following with lipid immunostaining (Bodipy). In the HP, we observed a significant increase in the intensity of Bodipy signal in HIV-Tg rats compared to controls implying HIV proteins could dysregulate lipid metabolism and increase LDs formation in this area (Figs. 1A and 1C, HIV-Tg rats vs. WT, unpaired two tail t-test, 2.33 ± 0.06 folds, * P = 0.0004). To identify the cellular origin of these increased LDs, we performed double immunostaining of ionized calcium-binding adapter molecule 1 (ba1, microglial marker) and Bodipy. There was a significant upregulation in Iba1 intensity in the HP of HIV-Tg rats compared to controls implying increased microglial activation (Figs. 1A and 1D, HIV-Tg rats vs. WT, unpaired two tail t-test, 1.31 ± 0.07 folds, *P = 0.0018). Interestingly, we also detected a high percentage of co-localization of LDs with Iba1 (Figs. 1A and 1F, ~ 70%) suggesting increased LDs are mainly from microglia (LDAM). To discern whether astrocytes also contribute to the increased LDs formation, we performed double immunostaining of glial fibrillary acidic protein (GFAP, astrocyte marker) and Bodipy. Although there is upregulation trend on GFAP intensity, the difference did not reach significance between these two strains of rats (Fig. 1B and 1E, HIV-Tg rats vs. WT, unpaired two tail t-test, 1.13 ± 0.08 folds, P = 0.1486). Also, there is only minimal overlapping of GFAP with LDs (Figs. 1B and 1F, ~ 5%). These results indicate that HIV proteins have marginal effects on lipid metabolism in astrocytes in the HP. Taken together, our results demonstrate that HIV proteins could dysregulate lipid metabolism and increase LDAM in the HP and increased LDs formation is associated with enhanced microglial activation.

**Figure 3.**
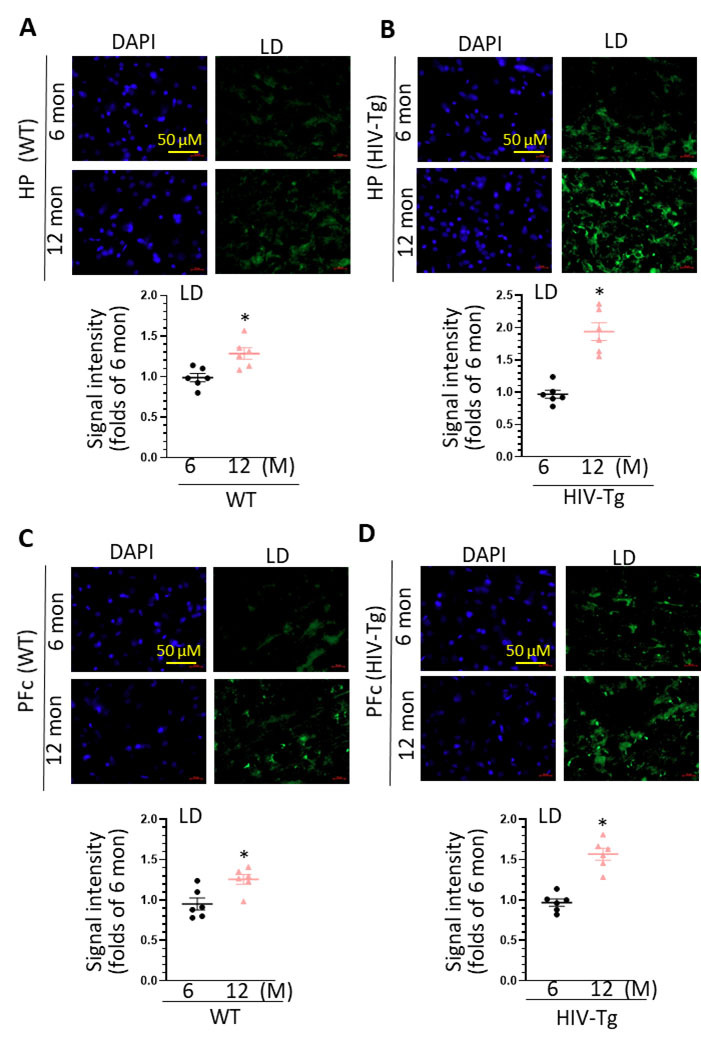
**LDs accumulation in the brains of WT and HIV-Tg rats during aging**. (**A**) increased LDs accumulation in the HP of WT rats during aging (scale bar = 50 µm, * P < 0.05); (B) increased LDs accumulation in the HP of HIV-Tg rats during aging (scale bar = 50 µm, * P < 0.05); (C) increased LDs accumulation in the PFc of WT rats during aging (scale bar = 50 µm, * P < 0.05); (D) increased LDs accumulation in the PFc of HIV-Tg rats during aging (scale bar = 50 µm, * P < 0.05). Each group contains 3 rats and two slices were selected from each rat (n = 6), unpaired two tail t-test was used for statistical analysis.

**Figure 4.**
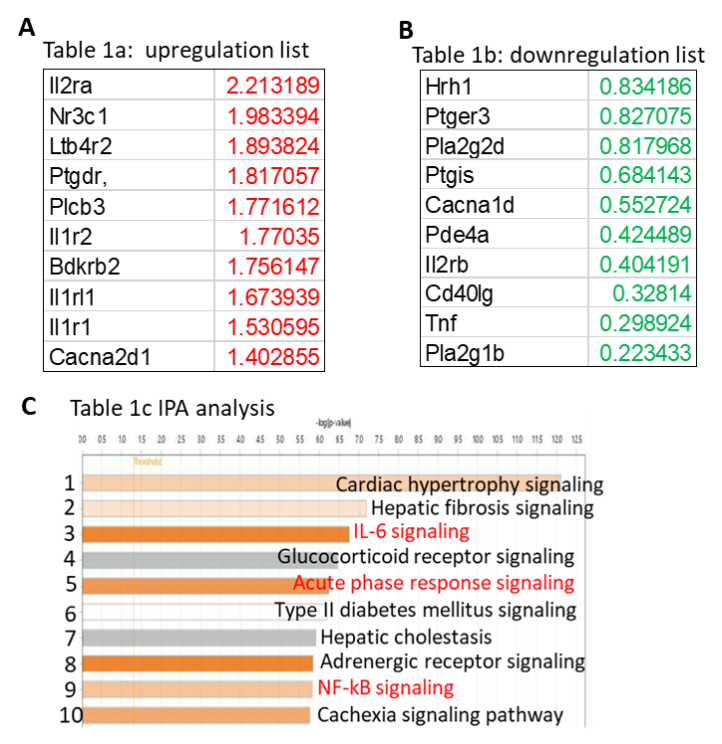
**Dysregulation on inflammation-related genes in microglia isolated from the HP of HIV-Tg rats**. (**A**) Top 10 genes with upregulation in microglia of HIV-Tg rats compared to WT counterparts; (B) Top 10 genes with downregulation in microglia of HIV-Tg rats compared to WT counterparts; (C) IPA analysis show top 10 pathways dysregulated in microglia of HIV-Tg rats.

### HIV-Tg rats show an increased LDs formation in astrocytes in the prefrontal cortex (PFc)

We next explored the effects of HIV protein on LDs formation in the PFc. Similarly, we observed a significant increase in the intensity of LDs in the PFc of HIV-Tg rats compared to controls (Figs. 2A and 2C, HIV-Tg rats vs. WT, unpaired two tail t-test, 2.37 ± 0.06 folds, * P = 0.0003). There is also a significant increase in Iba1 intensity (Figs. 2A and 2D, HIV-Tg rats vs. WT, unpaired two tail t-test, 1.19 ± 0.05 folds, * P = 0.0118). However, we did not observe increased GFAP levels in the PFc of HIV-Tg rats (Figs. 2B and 2E, HIV-Tg rats vs. WT, unpaired two tail t-test, 1.08 ± 0.07 folds, P = 0.5854). Surprisingly, there is a higher percentage of co-localization of GFAP with LDs than the co-localization of Iba1 with LDs (60% vs. 5%) (Fig. 2F). These data imply that increased LDs formation is mainly from astrocytes but not from microglia in the PFc. These findings suggest that lipid metabolism dysregulated by HIV proteins is in a region-specific manner.

### HIV-Tg rats showed an accelerated LDs accumulation in the brains during aging

We have revealed significant LDs accumulation in the brains of HIV-Tg rats at middle age. We next investigated whether such an increase could happen at an earlier age. The brains of HIV-Tg and control rats (both genders, 6-month, n = 3) were removed out for cryo-section following with Bodipy staining. In the HP, we observed an upregulation trend in LDs signal in HIV-Tg rats, but such an increase did not reach statistical significance (Supplementary Fig. 1A, HIV-Tg rats vs. WT, unpaired two tail t-test, 1.17 ± 0.09 folds, P = 0.08). However, there is significant upregulation on LDs in the PFc of HIV-Tg rats (Supplementary Fig. 1B, HIV-Tg rats vs. WT, unpaired two tail t-test, 1.31 ± 0.11 folds, * P = 0.02). It is well-known that LDs accumulate during aging. To explore whether HIV proteins could affect that process, we compared LDs formation of these two strains at two different ages (12 vs. 6 month). In WT rats, there is a significant upregulation on LDs formation in the HP during aging (Fig. 3A, WT rats, 12 mon vs. 6 mon, unpaired two tail t-test,1.29 ± 0.10 folds, * P = 0.02); while in HIV-Tg rats, three is a higher upfolds on LDs formation in the HP (Fig. 3B, HIV-Tg rats, unpaired two tail t-test,12 mon vs. 6 mon, 1.96 ± 0.18 folds, * P = 0.0008). We observed a similar phenomenon on LDs formation in the PFc. The increased folds in WT and HIV-Tg rats are 1.29 ± 0.12 folds and 1.62 ± 0.10 folds, respectively (Figs. 3C and 3D, 12 mon vs. 6 mon, unpaired two tail t-test, * P < 0.05). Taken together, these results demonstrate that lipid metabolism dysregulation could happen at earlier age of HIV-Tg rats with various sensitivity in different regions and HIV proteins could accelerate LDs formation during aging. These findings imply that dysregulation on lipid metabolism is an early event on the pathogenesis of HAND.

**Figure 5.**
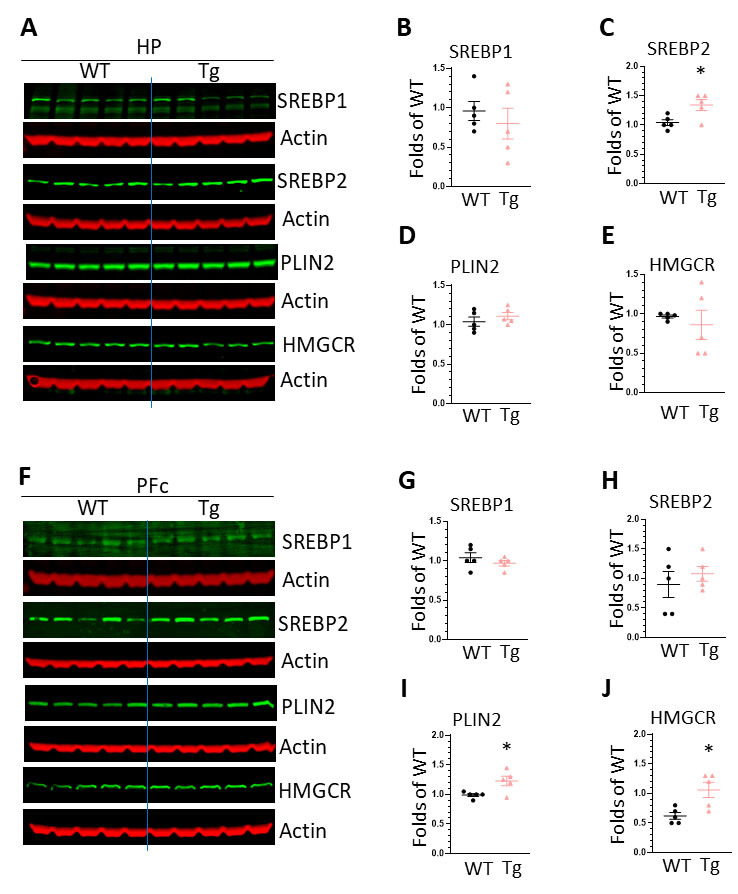
**The expression of molecules regulating cholesterol synthesis in the brains of WT and HIV-Tg rats**. (**A**) WBs images showing the expression of SREBP1, SREBP2, PLIN2, HMGCR in the HP of WT and HIV-Tg rats; (B) statistical analysis of SREBP1 levels in the HP of WT and HIV-Tg rats; (C) statistical analysis of SREBP2 levels in the HP of WT and HIV-Tg rats (* P < 0.05); (D) statistical analysis of PLIN2 levels in the HP of WT and HIV-Tg rats; (E) statistical analysis of HMGCR levels in the HP of WT and HIV-Tg rats; (F) WBs images showing the expression of SREBP1, SREBP2, PLIN2, HMGCR in the PFc of WT and HIV-Tg rats; (G) statistical analysis of SREBP1 levels in the PFc of WT and HIV-Tg rats; (H) statistical analysis of SREBP2 levels in the PFc of WT and HIV-Tg rats; (I) statistical analysis of PLIN2 levels in the HP of WT and HIV-Tg rats (* P < 0.05); (J) statistical analysis of HMGCR levels in the HP of WT and HIV-Tg rats (* P < 0.05). Each group contains 5 rats, unpaired two tail t-test was used for statistical analysis.

### Adult microglia from HIV-Tg rats show dysregulated neuroimmune signaling

To identify genes and pathways underlying the increased microglial activation, we isolated adult microglia from HIV-Tg and WT rats (n = 3). After the validation (> 90% cells are Iba1 immunostaining positive), total RNAs were extracted for inflammation-enriched gene array analysis. Among the eighty-four genes listed in the array, the top 10 genes with the most up- or down- regulation based on fold changes were shown in Figure 4A and B, respectively. Among the upregulated genes, interleukin 2 receptor subunit alpha (il2ra, 2.21 folds), interleukin 1 receptor type 2 (il1r2,1.77 folds), interleukin 1 receptor-like 1 (il1rl1, 1.67 folds), and interleukin 1 receptor type 1 (il1r1, 1.53 folds) are belonged to the superfamily of interleukin receptors which is well-known for regulating immune responses [51]. Nuclear receptor subfamily 3 group C member 1 (Nr3c1) encodes glucocorticoid receptor which is in response to stress and regulates immune response by modulating availability of stress hormone cortisol [52]. Among the downregulation genes, phospholipase A2, group 1B (Pla2g1b, 0.22 folds) and Pla2g2d (0.81 folds) encode proteins belonged to phospholipase family which modulates fatty acid and cholesterol metabolism. Indeed, the activity of lipase could modulate LDs formation in microglia [53]. Overall, the alterations on gene expression in adult microglia suggest increased microglial activation and lipid metabolism dysregulation. IPA revealed the top ten pathways dysregulated in HIV-Tg microglia (Fig. 4C). As observed, pathways including Il6 signaling, acute phase response signaling, and nuclear factor kappa B (NF-кB) signaling are upregulated consolidating microglia are in activate state.

**Figure 6.**
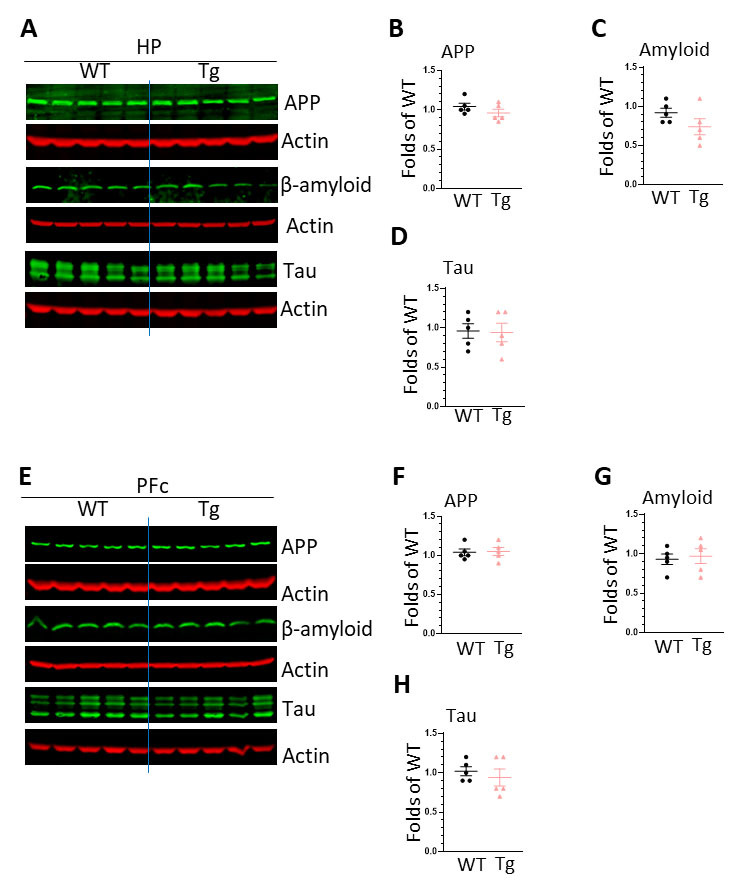
**The expression of neurodegeneration markers in the brains of WT and HIV-Tg rats**. (**A**) WBs images showing the expression of APP, β-amyloid, Tau in the HP of WT and HIV-Tg rats; (B) statistical analysis of APP levels in the HP of WT and HIV-Tg rats; (C) statistical analysis of β-amyloid levels in the HP of WT and HIV-Tg rats (* P < 0.05); (D) statistical analysis of Tau levels in the HP of WT and HIV-Tg rats; (E) WBs images showing the expression of APP, β-amyloid, Tau in the PFc of WT and HIV-Tg rats; (F) statistical analysis of APP levels in the HP of WT and HIV-Tg rats; (G) statistical analysis of β-amyloid levels in the HP of WT and HIV-Tg rats (* P < 0.05); (H) statistical analysis of Tau levels in the PFC of WT and HIV-Tg rats. Each group contains 5 rats, unpaired two tail t-test was used for statistical analysis.

**Figure 7.**
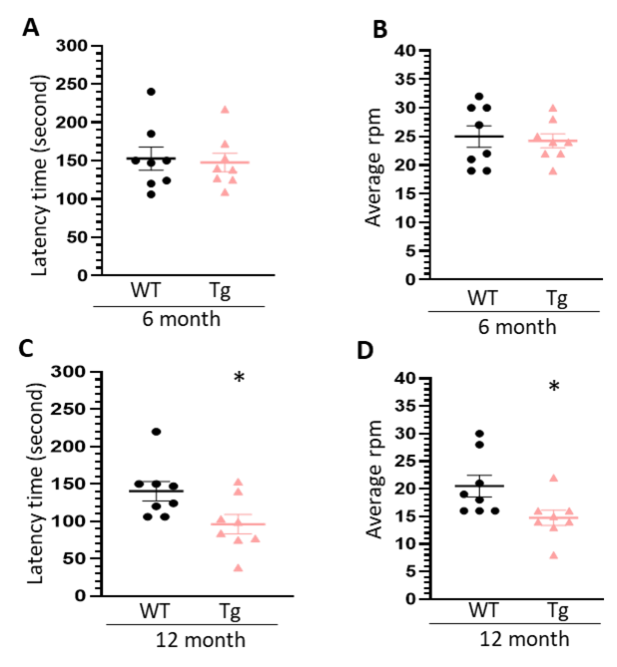
**Rotarod tests for WT and HIV-Tg rats during aging**. (**A**) statistical analysis of latency time of WT and HIV-Tg rats in 6-month-old in rotarod tests; (B) statistical analysis of speed (round per minutes, rpm) of WT and HIV-Tg rats in 6-month-old in rotarod tests; (C) statistical analysis of latency time of WT and HIV-Tg rats in 12-month-old in rotarod tests; (D) statistical analysis of speed (round per minutes, rpm) of WT and HIV-Tg rats in 12-month-old in rotarod tests. Each group contains 8 rats, unpaired two tail t-test was used for statistical analysis.

### HIV-Tg rats show increased activity of cholesterol synthesis

We next explored the mechanisms underlying increased LDs formation and firstly focused on cholesterol synthesis pathway. Thus, SREBP1/2, HMGCR, and perilipin-2 (PLIN2, LDs formation marker) were selected and their expression levels were determined in the HPs and PFcs of HIV-Tg and WT rats (middle age, n = 5). In the HP, HIV-Tg rats showed an increase in the expression of SREBP2 (Figs. 5A and Fig. 5C, HIV-Tg rats vs. WT, unpaired two tail t-test, 1.42 ± 0.10 folds, * P < 0.05) but no increase on SREBP1, PLIN2, HMGCR (Figs. 5B, 5D, 5E, HIV-Tg rats vs. WT, unpaired two tail t-test, P > 0.05). In the PFc, HIV-Tg rats show a significant increase in PLIN2 and HMGCR (Figs. 5I and 5J, HIV-Tg rats vs. WT, unpaired two tail t-test, * P < 0.05). There is an upregulation trend on SREBP2 levels in the HIV-Tg rats but did not reach statistical significance (Fig. 5H, HIV-Tg rats vs. WT, unpaired two tail t-test, P > 0.05). Overall, we observed an increasing activity on cholesterol synthesis in HIV-Tg rat brains. Another possible mechanism for upregulation on LDs formation is lipophagy defection. We checked the levels of autophagy-related molecules including beclin1 (marker for autophagy initiation), p62 (index for lysosome degradation ability), and LC3B (autophagy marker) in the brains of HIV-Tg and WT rats. There are no significant changes on the levels of these three molecules between HIV-Tg and WT rats (Supplementary Figs. 2A - 2H, HIV-Tg rats vs. WT, unpaired two tail t-test, P > 0.05). Taken together, these findings suggest that increased LDs formation in HIV-Tg rats is mainly due to the enhanced cholesterol synthesis but not from lipophagy defection. Increased LDs formation and subsequent microglial activation can be linked with neuronal injuries and improper protein misfolding and accumulation. Beta-amyloid accumulation and tubulin associated unit (Tau) protein aggregates are markers for aging and neurodegeneration. Therefore, we checked the levels of amyloid precursor protein (APP), β-amyloid, and Tau in the brains of HIV-Tg and WT rats. There are no significant changes on the levels of these three molecules (Figs. 6A - 6H, HIV-Tg rats vs. WT, unpaired two tail t-test, P > 0.05). These results suggest that LDs formation and microglial activation precede neuronal pathology in rodent models of HAND. In supporting this, we also monitored postsynaptic density protein 95 (PSD95) levels (marks of excitatory synapse) in the brains and found no significant difference between these two strains (data not shown here).

### HIV-Tg rats reveal deficiency on locomotion coordination

The gradual decline in the ability of locomotion coordination is a reflection on decreased physiological function during the aging process. To investigate whether HIV-Tg has accelerated decline on this ability, we performed rotarod tests on HIV-Tg and WT rats at 6 and 12 months (both genders). At 6-month-old, there is a decreased trend but no significant difference on the latency time and average rotation speed before falling between HIV-Tg and WT rats (Figs. 7A, 7B, HIV-Tg rats vs. WT, unpaired two tail t-test, n = 8). However, at middle age, HIV-Tg rats showed a significant decrease on the latency time as well as average speed on rotarod before falling (Figs. 7A, 7B, HIV-Tg rats vs. WT, unpaired two tail t-test, n = 8, P < 0.05). These data demonstrated that HIV-Tg rats have impairment on locomotion coordination implying accelerate aging process compared to WT controls.

## DISCUSSION

Chronic HIV (+) individuals show accelerated brain aging with higher incidence on neurological symptoms compared to age-matched HIV negative population [4, 5]. Abnormal microglial activation plays critical roles in accelerating brain aging. Defections on multiple signaling pathways including autophagy, inflammasome, TLR/NF-кB, and lysosome have been suggested to contribute to microglial activation in the context of HIV infection *in vitro* and *in vivo* [54-56]. Recently, accumulating evidence indicate that lipid/cholesterol metabolism is heavily involved in microglial activation [20-22]. LDAM, a novel phenotype of microglia identified in aging brain and neurodegenerative disease, could induce neuronal injuries through increased secretion of pro-inflammatory mediators and cytokines as well as phagocytosis defection. Lipid metabolism dysregulation (LDs formation) has been shown as an early event for microglial activation and brain aging. In this study, in order to explore the involvement of lipid metabolism in accelerated aging in the context of HIV infection, we compared the status of LDs formation in the brains of HIV-Tg and WT rats at two different ages. Our findings demonstrate the positive correlation between LDs formation, microglial activation, and behavioral impairment in HIV-Tg rats. These results add a new layer of complexity of mechanisms (immunometabolism) responsible for microglial activation and accelerated brain aging induced by HIV infection. Currently, there are no effective treatments for ameliorating HAND symptoms in clinics. These findings indicate that restoration of cholesterol or lipid metabolism could be a novel therapeutic approach to inhibit microglia and improve HAND symptoms.

### The selection on animal model

For investigating the mechanisms underlying accelerated aging and HAND in the context of HIV infection, several rodent models are available including HIV inducible (i) TAT mice [57], HIV-gp120 mice [58], HIV-Tg26 (FVB) mice [59], and HIV-Tg rats [60]. Each model has been developed to represent one facet of HAND pathogenesis with both advantages and disadvantages. HIV-iTAT and HIV-gp120 mice only express one specific HIV protein (TAT or gp120) in the brain and they are suitable for investigating the effects of one certain HIV protein on neuroinflammation and neuronal injuries *in vivo*. For HIV-Tg26 (FVB) mice, their genetic background could lead to severe kidney pathology during aging and most mice are moribund at the age of 6 month [59]. After careful consideration, we selected HIV-Tg rats in our studies: (1) HIV-Tg rats show relative healthy status in the first six month and are gradually evident on pathological changes in the middle age; (2) HIV-Tg rats express seven of nine HIV proteins *in vivo* which better mimic the scenario happened in chronic HIV (+) individuals on cART [60]; (3) Previous report already revealed HIV-Tg rats with dysregulated lipogenic genes on cART [61]. The disadvantage for this model is that HIV-Tg rats are somewhat blind to the surroundings due to the cataract. So, HIV-Tg rats are not suitable for performing memory and cognitive tests such as novel object recognition and elevated maze tests. We notice that there is a newly developed mouse model HIV-Tg26 with C57BL/6j background. These mice can have prolonged life expectancy with relative health status and have been used for aging studies [62]. In the future, we would expand our studies by using HIV-Tg26 mice to validate our hypothesis with more behavioral analysis.

### Lipid metabolism dysregulation and HAND pathogenesis

Accumulating evidence demonstrates that various inflammatory stimuli including HIV proteins can dysregulate lipid and/or cholesterol metabolisms leading to LDs accumulation in the brain. LPS could increase LDs formation in microglia *in vitro* and *in vivo* [28, 44]. HIV-TAT has been shown to dysregulate cholesterol homeostasis in neurons and astrocytes which is linked with HAND pathogenesis [45, 46]. HIV-Nef has the ability to dysregulate cholesterol metabolism and lipid raft *in vivo* [48-50]. However, the direct evidence showing the effects of HIV proteins on microglial lipid metabolism *in vivo* remains much scarce. In this study, we demonstrated increased LDs formation in the brains of HIV-Tg rats at middle age comparing WT counterparts. Interestingly, LDs are more evident in microglia in the HP but are more co-localized with astrocytes in the PFc. These results indicate that HIV proteins could dysregulate lipid metabolism both microglia and astrocyte in a regions specific manner, however, the underlying mechanisms remain elusive. In HIV-Tg rats, hippocampal microglia show enhanced activation indicated by the increased Iba1 intensity as well as the results obtained from inflammatory gene array analysis. We observed a positive association between LDs formation and microglial activation which is consistent with previous reports. On the contrary, we did not detect increased astrocyte activation in the PFc since there is no upregulation on GFAP levels in HIV-Tg rats. Such differential effects may be due to the difference on the primary function for microglia and astrocytes *in vivo*. Microglia are brain residential macrophages and can be easily activated by multiple external and internal stimuli while the primary function of astrocyte is to provide energy or nutrient to support for brain and neuronal homeostasis. The increased LDs formation in astrocytes imply the possible defection on lipid/cholesterol efflux which may cause disruption on brain and neuronal functions, a mechanism underlying the pathogenesis of HAND [46]. Another possibility is that astrocyte activation is a late event during aging and can only be observed in advanced age of HIV-Tg rats. We can test this assumption using more advanced age of HIV-Tg rats (18 month).

Another interesting finding is the speedup of LDs accumulation in HIV-Tg rats during aging. At mature age (6 month), there is already an increase on LDs formation in the brains of HIV-Tg rats comparing to WT (1.17 ± 0.08 folds in the HP and 1.31 ± 0.11 folds in the PFc). However, during normal aging, HIV-Tg rats showed the accelerated speed on LDs formation in the brains. In the HP, WT rats showed 1.29 ± 0.10 folds upregulation (12 month vs. 6 month) while HIV-Tg rats reveal 1.96 ± 0.18 folds increase. Similarly, in the PFc, WT and HIV-Tg rats have 1.29 ± 0.12 and 1.62 ± 0.10 folds upregulation, respectively. Accompanying with this increased LDs accumulation, HIV-Tg rats showed defection on locomotion coordination on rotarod tests. These results suggested that increased LDs formation in the brain may be associated with accelerated aging process in the context of chronic HIV infection. Our findings are consistent with previous report showing that LDAM play critical roles in promoting aging process [22]. Although HIV infection has been long known for dysregulating lipid metabolism in the peripheral system, direct evidence on its effects on central nervous system especially in microglia are very scarce. Our findings, for the first time, provide solid evidence that HIV proteins could directly dysregulate lipid metabolism in microglia. In addition, we identified increased LDs formation in astrocytes in the PFc during aging. Whether LD accumulation in astrocytes could lead to astrocyte dysfunction as well as neurological symptoms would be an interesting topic in further investigation.

### Mechanisms underlying LDs formation

LDs are spherical organelles that store intracellular neutral lipids such as triacylglycerols and cholesteryl esters. LDs are dynamic organelles and the formation is regulated by multiple process including the synthesis, influx/efflux traffic, and degradation (lipase, and autophagy) [32]. To explore the mechanism underlying the increased LDs formation, we explored the activity of lipid synthesis and autophagy pathways in the brains of WT and HIV-Tg rats. We found a significant increase on SREBP2 levels in the HP and also for PLIN2 and HMGCR in the PFc of HIV-Tg brains implying enhanced synthesis activity on cholesterols promoting LDs formation. On the contrary, we did not find significant difference on the levels of beclin1, p62, and LC3II between these two strains suggesting that autophagy process is not compromised in the brains of HIV-Tg rats. Therefore, we argue that HIV proteins could probably affect the synthesis pathway but not degradation process leading to the increased LDs formation *in vivo*. One limitation of our method is that we employed brain homogenates to monitor autophagy process. Due to the fact that microglia only account for 10 - 15% of brain cells, the subtle but significant changes on microglial autophagy might be cloaked. We will use purified microglial for interrogating autophagy in future studies. Tau protein aggregates and β-amyloid accumulation are the markers for neurodegeneration [63, 64]. We also checked the levels of APP, β-amyloid, and Tau in the brains of HIV-Tg and WT rats and found no significant differences. These results indicate that changes in lipid metabolism and microglial activation precede neuronal dysfunction, adding more evidence to support that LDs formation is an early event during aging and neurodegeneration.

### Limitations on our studies

Our findings show that HIV proteins could dysregulate lipid metabolism in cell- and region-specific manners during the aging process. There are some limitations in our studies. This is a description of the investigation. Since multiple HIV proteins such as TAT, gp120, Nef, etc., could be expressed in the brain so we could not discern which protein(s) exert(s) such dysregulation on lipid metabolism or whether the presence of the several proteins together is necessary for such dysregulation. *In vitro* mechanism study (cell culture) or using another rodent model only expressing one HIV protein (for example, HIV-iTAT mice) could answer these questions. We included both genders of rats in our study but did not stratify the data from male and female rats within groups (small size). There could be possible gender differences on LDs formation in HIV-Tg rats and this could be answered by adding more rats in future study.

In conclusion, we demonstrated that HIV proteins are capable of increasing LDs formation in region- and cell-type specific manners in the brain. Such dysregulation is positively associated with microglial activation and accelerated aging. These results indicate that restoration on lipid metabolism could be a novel therapeutic target for ameliorating the accelerate aging and HAND symptoms in chronic HIV infection individuals.

## Supplementary Materials

The Supplementary data can be found online at: www.aginganddisease.org/EN/10.14336/AD.2024.0125
