# Supplementary material for: Lipid Droplets Accumulation in the Brain of HIV Transgenic Rat: Implication in the Accelerated Aging of HIV Infected Individuals
Source: Aging Dis. 2024 Jan 25;16(1):454–68. doi: 10.14336/AD.2024.0125 (PMC11745453; doi:10.14336/AD.2024.0125)
Supplement: Supplementary file 1 — The Supplementary data can be found online at: www.aginganddisease.org/EN/10.14336/AD.2024.0125 [file AD-16-1-454-s.pdf]

SUPPLEMENTARY DATA

Lipid Droplets Accumulation in the Brain of HIV Transgenic Rat: Implication in the Accelerated Aging of HIV Infected Individuals

Ming-Lei Guo, Yan Cheng, Damian Martinez Pineda, Rachael E. Dempsey, Lifang Yang

© 2024. Guo ML et al. Published online at http://www.aginganddisease.org/EN/10.14336/AD.2024.0125

SUPPLEMENTARY DATA

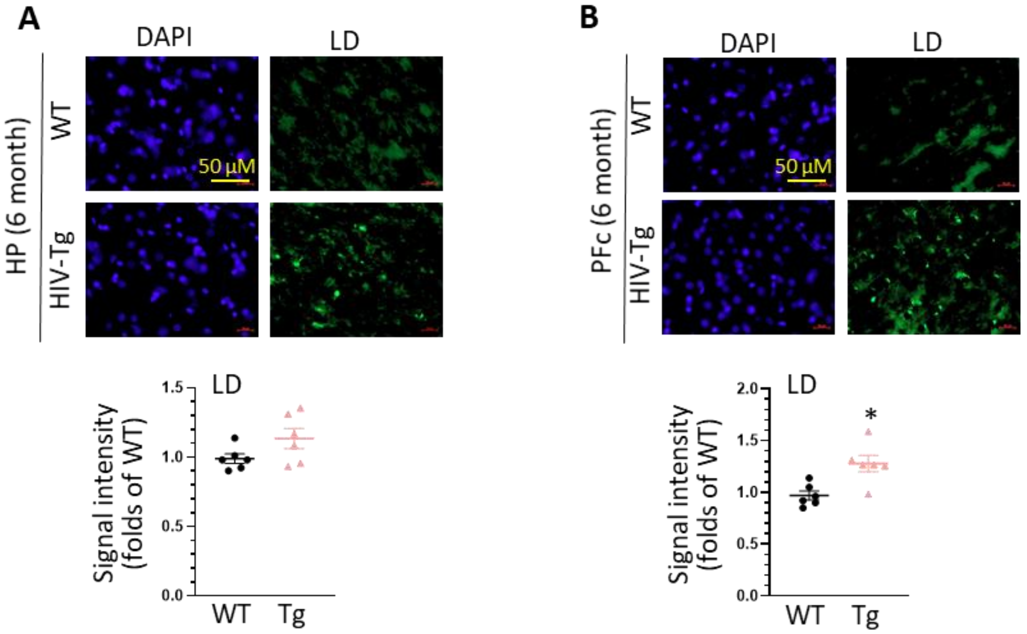

Supplementary Figure 1. LDs accumulation in the brains of WT and HIV-Tg rats at 6-month old: (**A**) LDs accumulation in the HP of WT and HIV-Tg rats (scale bar = 50 µm, P = 0.08); (**B**) LDs accumulation in the PFc of WT and HIV-Tg rats at 6-month old (scale bar = 50 µm, * P < 0.05); Each group contains 3 rats and two slices were selected from each rat (n = 6), unpaired two tail t-test was used for statistical analysis.

© 2024. Guo ML et al. Published online at http://www.aginganddisease.org/EN/10.14336/AD.2024.0125

SUPPLEMENTARY DATA

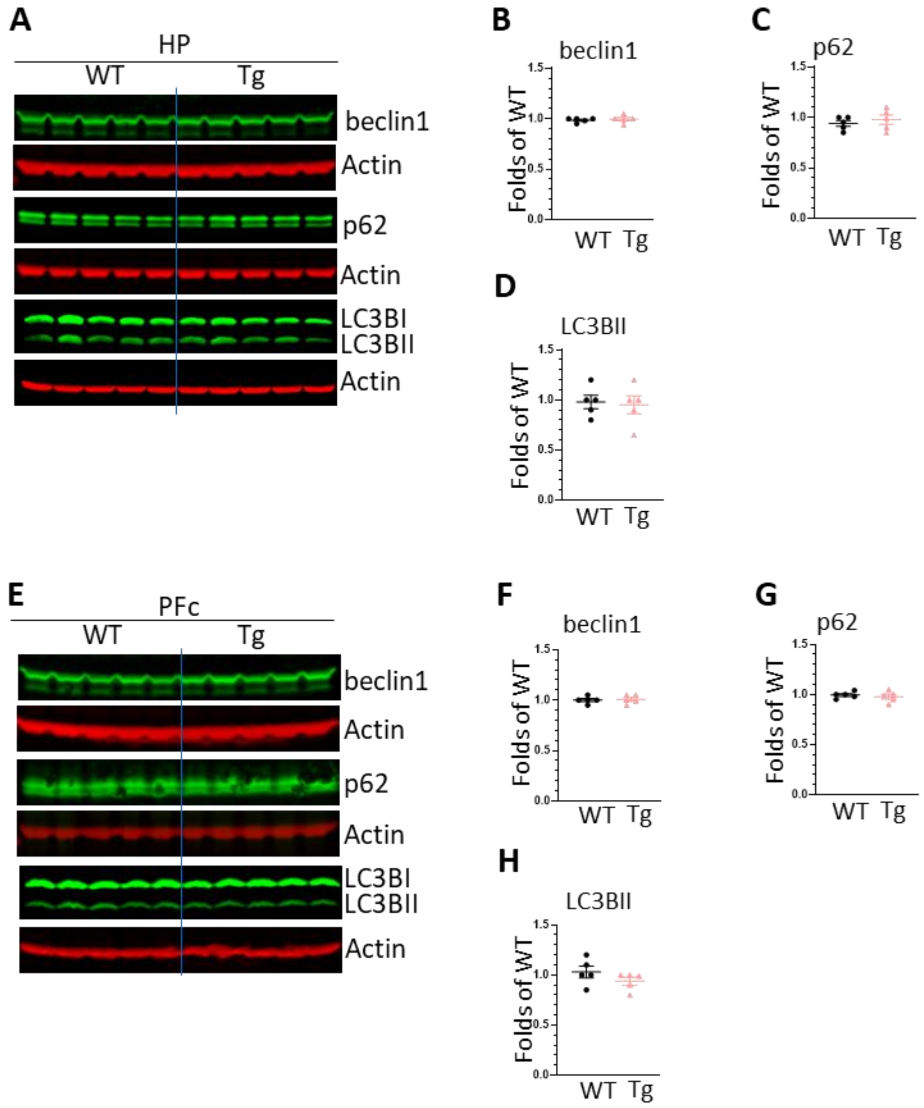

Supplementary Figure 2. The expression of autophagy-related molecules in the brains of WT and HIV-Tg rats (12 month old): (**A**) WBs images showing the expression of beclin1, p62, and LC3B in the HP of WT and HIV-Tg rats; (**B**) statistical analysis of beclin1 levels in the HP of WT and HIV-Tg rats; (**C**) statistical analysis of p62 levels in the HP of WT and HIV-Tg rats; (**D**) statistical analysis of LC3BII levels in the HP of WT and HIV-Tg rats; (**E**) WBs images showing the expression of beclin1, p62, LC3B in the PFc of WT and HIV-Tg rats; (**F**) statistical analysis of beclin1 levels in the PFc of WT and HIV-Tg rats; (**G**) statistical analysis of p62 levels in the PFc of WT and HIV-Tg rats; (**H**) statistical analysis of Tau levels in the PFC of WT and HIV-Tg rats. Each group contains 5 rats, unpaired two tail t-test was used for statistical analysis.

© 2024. Guo ML et al. Published online at http://www.aginganddisease.org/EN/10.14336/AD.2024.0125
